# Sedentary Behaviour among Urban Civil Servants in Eastern Part of Southern Nations, Nationalities and Peoples' Region, Ethiopia

**DOI:** 10.1155/2021/8847107

**Published:** 2021-03-22

**Authors:** Markos Yohannes Baye

**Affiliations:** Department of Sports Science, College of Natural and Computational Sciences, Dilla University, Ethiopia

## Abstract

**Background:**

Active lifestyle is a determining factor for functional and clinical health that protects and maintains both physical and mental health of an individual, whereas sedentary lifestyle is a contrary vital cause for higher premature mortality, heart disease, diabetics, and poorer quality of life. This study is aimed at determining the amount of time spent on sedentary activity and identifying sedentary behaviours frequently practiced by civil servants in Southern Ethiopia in 2015.

**Methods:**

It was a cross-sectional study which employed both qualitative and quantitative approach. A stratified cluster sampling method was used to select 375 office workers (222 men and 153 women) from Hawassa, Wolayta Soddo, and Dilla ranging from 18-65 years old. Data were collected using harmonized self-reporting LASSA (Longitudinal Aging Study Amsterdam) questionnaires and prevalence estimates of mean sedentary time in each 12 activities per day were determined. Descriptive and inferential statistics such as Independent *t*-test, Uni-variate ANOVA, and Person's correlation were used to analyze association and predictability of IV on DV variables.

**Result:**

The total mean time spent sitting per day was 13.39 h which was 81.5% of weak time. Collectively, screen time was dominant (6.08). About 70.7%, 23.7%, 4.8%, and 0.8% of respondents were levelled very high, high, moderate, and less sedentary, respectively. In general, women accounted higher sedentary level (96.1%) than men (93.3%) in sedentary activity. There is a weak positive correlation between age and time spent in an administrative task. Income and mealtime were statistically significant (*r* < 0.2, *n* = 375, *p* < 0.05).

**Conclusion:**

The high level of self-reported sedentary time record suggests the need for public health policies targeted at increasing physical activity and decreasing sitting time through systemic intervention in and out of work.

## 1. Background

As active lifestyle is a determining factor for functional and clinical health, protect and maintain both physical and mental health of an individual, whereas sedentary lifestyle is a contrary vital cause for higher premature mortality, heart disease, diabetics, and poorer quality of life [1]. Evidence reveals that sedentary behaviour (SB) is exposing its harmful health effect in the contemporary population [2]. Alarm ables warning for sedentary individuals saying: “Are are you sitting down? It's slowly killing you. Regular workouts don't decrease death risk if you're also a couch potato”; “Sitting Too Much Could be Deadly”; “Those with a desk job, please stand up” were emerging phenomenon. Meaning that SB is independent of physical activity (PA), and active people who meet the recommended level of PA or even who achieve a high level of PA can be sedentary if you sit too much time [3]. Individuals can be both sedentary and inactive as there is also potential for high sedentary time and high exercise time to coexist [4]. WHO on its report of (2010) demonstrated that physical inactivity and obesity become the leading risk factors for global mortality. The numbers of people who pass away each year reaches 3.2 million due to physical inactivity because people who are sedentary have a 20% to 30% greater likelihood of death in any case compared with active people [5]. In contrary, proper PA evens against illness or death (morbidity motility) [1]. The association of SB with deleterious health hazards has been explored by the prominent sedentary researcher Biddle et al. For example, those who are viewing TV for more than 2 hours a day have been found having adverse body composition, decreased fitness, lowered self-esteem and pro-social behaviour, and reduced academic achievement [6]. Also, Beckford stated, watching too much TV is as dangerous as smoking or being overweight [7]. Systematic Studies held by Costigan et al., (2012) found that spending time sitting in front of a screen for greater than 4 hours a day has magnificent adverse health effects on an individual [8]. Screen time (the time spent watching TV and movies, playing video games, and using computers) accounts for the majority share of time per day spent in sedentary which is negatively associated with multiple adverse functional and medical health outcomes [9, 10]. Medical researchers have long warned that prolonged sitting in a dangerous office chair is worse for your health than smoking and kills more people than HIV [11]. According to (ACSS) American Cancer Society Study, women who were inactive and sat over six hours a day were 94% more likely to die during the time period studied. SB and life expectancy in the USA: a case analysis by Peter Katzmarzyk as reported by Ergotron, population life expectancy would increase two years if adults reduce their sitting time by at least three hours per day [12].

There are many opportunities to sit in our daily lives, there is no running away from it, the key is to find opportunities to move [12, 13]. Modern research definition of SB rejects the approach of lack of PA. Instead, it favours the behaviours performed while in the position of sitting or lying in which the energy expenditure is low, which means that the energy expenditure level is 1.0-1.5 metabolic equivalent (METs) where 1 MET is equivalent to the energy cost of quit rest [4, 14, 15]. Even though still, there are no recommended cut points established for SB definition, recent literature revealed to define sedentary with respect to hours spent per day. Sitting or reclining at work and at home; getting to and from places; time with friends and time spent sitting at a desk; travelling in car, bus, and train; reading, playing cards, or watching television; etc. except time spent sleeping are SB. SB can be categorized into three levels, called “low,” “middle,” and “high” corresponding (2.5 h/day), (5 h/day), and (10 h/day), respectively [16]. Moreover, the most recent works came up with a new approach to explain SB having precise justification. In view of that, due to the fact that impracticality of measuring energy expenditure in most studies and due to the existence of some limited behaviours that performed while sitting but energy expenditure is (>1.5 METs) [17–19].

Historically, SB is increased with the emergence of technological innovations and industrialization [20]. The consequence of this technological and industrial revolution became lifestyle change, which had a significant impact on decreasing physical endeavor in daily life and had encouraged sedentary lifestyles among both young people and adults over the past 2 to 3 decades [4, 21]. For example, in the work of Al-Nakeeb et al., it is indicated that in recent decades, majority of Arab cities have shown remarkable lifestyle changes due to fast urbanization. Studies showed a dramatic decrease in jobs requiring moderate physical activity in the US from 50% to 20% within 5 decades. In the early 1960s, half of the jobs were requiring physical challenge, but in 2008, such jobs decreased to 20% [22]. Fox also reported that “with the emergence of technological advancement, miss-match between the food availability (food intake) and pursue to access food (energy expenditure) resulted in new pandemic of obesity, type 2 diabetics and the likes in the UK” [3]. This is contemplation or an exhibit of how PA reduced as modern years increased [22].

Inactivity or little PA and sitting too much has diverse physiological effects epidemiologically investigated the correlation with cardio-metabolic functions. This contemporary evidence showed that sedentary physiology called “inactive physiology” is quite different from “exercise physiology” in their biological mechanism [4, 23]. The pioneering work of Hamilton and colleagues reported that (as cited in Owen et al., 2010) a prolonged period of muscular inactivity is associated or similar with extended sedentary time leads to inhibition of skeletal muscle lipoprotein lipase (LPL) activity, which is very important for triglyceride uptake and high-density lipoprotein (HDL) cholesterol production, and decreased glucose consumption have deleterious biological hazards [4]. Decreased levels of (HDL) cholesterol and decreased insulin sensitivity are the most important characteristics of metabolic dysfunction [23].

The prevalence rate of SB was studied and demonstrated a number of pieces of evidence, particularly among developed countries. For example, Spittaels et al. reported that 57% of US (7.89 h/a day), 55% of Sweden (7.7 h/a day), 57% of Australian (8.12 h/a day), 58% of European adults (8.12 h/a day), and 58% of western countries (7.89 h/a day) dedicated their waking time in sedentary pursuit [24]. For example, in the 21 years follow-up examination in the US, it reported that those sitting in automobiles more than 10 h a week were compared with those spending less than 4 h a week had an 82% greater risk of dying from CVD [4].

Hence, numerous studies recommend Moderate Vigorous Physical Activity (MVPA) regardless of age and sex and reduce or break sitting time. However, still “at present, no definitive recommendations can be made on how long adults should sit for or how often they should break up their sitting time” [4, 10], breaking up sedentary time can be beneficial [4] but how often break up is remained to be answered by contemporary researchers. The health risk of SB has been started to be explored through research and reported that unlike exercise and diet, SB has the potential to determine or predict the future health status of people just as bad habits such as smoking [3]. As a result, countries are developing guidelines and recommending PA at least 30 min of MIPA per day, or 150 minutes of moderate-intensity aerobic exercise per week in multiple short bouts not less than 10 minutes or 75 min of VIPA or equivalent combination of MVIPA. Those who fail to meet these criteria were considered to be sedentary [1, 15, 25]. As Fox mentioned, the future health status of the people will be in danger if we failed to intervene or ignorant to aware and to take the necessary measurement [3]. Therefore, due to the public risks associated with largely negative consequences and potentially high prevalent rates, public health guidelines that recommend participation in PA and limiting SB have been produced by a number of countries [26]. But yet, almost nothing or very little is known about the effects and prevalence of SB in developing countries like Ethiopia. Though sitting too much is a global problem, more victimized are unaware and yet not starting to consider sitting too much as a risk factor for various public health problems and yet not intervene or develop intervention strategies to reduce too much sitting. Ethiopia is one of those African countries neither started intervention SB nor developed guidelines for PA recommendations.

Hence, there is a need to explore how adults spent time in their natural setting. Therefore, this study is aimed at ascertaining the prevalence, the time spent sedentary in sedentary activities, the level of SB, and its association with sociodemographic variables among civil servants in SNNPR, Ethiopia.

## 2. Methods

### 2.1. Study Design and Research Questions

The present study is an observational study in which a naturalistic observation survey merely used to collect descriptive information, namely, cross-sectional survey study conducted in between July and September in the year 2015 in Ethiopia is aimed at investigating the following research questions:

- For how long do office-based workers spent sedentary per day?
- What are the prevailing SB they engaged and practiced frequently?
- Are there any difference between men and women in sedentary practice?
- In which category of sedentary level most civil servants can be levelled?
- How it seems the relationship between dependent & independent variables (IV)?

Both qualitative and quantitative approaches were employed to explore sedentary time oddity, which is not yet experienced among the participant due to limited or no research endeavor in the current concept.

### 2.2. Study Area

Ethiopia is a federal government operating as nine decentralized States, and South Nation Nationalities People Region (SNNPR) is one of the nine states situated in the southern part of the country. SNNPR is also subdivided into 14 administrative geopolitical zones and 4 special woredas [27]. The study was conducted in three densely populated Zonal Towns situated in the Eastern part of the region namely Hawassa, Wolayta Soddo, and Dilla.

### 2.3. Study Participant

The participants were permanent (full time) employee of civil servants (adults) aged 18-65 years old who are engaged in office-based works in the aforementioned three towns in Governmental organizations. Governmental organizations in the region are structured in 14 administrative zones and municipalities, 1 regional bureau and Hawassa municipality, and 4 special woredas each containing 38 offices [27]. Particularly, Hawasa is a regional City, which comprises Sidama Zone sector offices, Zone municipality, and Regional bureau and Hawassa City musicality which accounted for 82.1% of the study population.

### 2.4. Sampling Strategy and Sample Size Determination

A stratified cluster random sampling method was employed to select 375 representative participants from 24,237 the total size of the target population residing in three towns proportional to the population size of stratum (residing Town). Sample size was determined by the use of Rao sample size calculating software which was an online survey conducting method used to estimate sample size [28] that is equivalent to the result from the formula *s* = *X*^2^ *NP*(1 − *P*) ÷ *d*^2^ (*N* − 1) + *X*^2^*P*(1 − *P*) used [29, 30]. The amount of error can be tolerated, that is, with a margin error of 5%, 95% confidence level, and 50% response distribution [30]. Accordingly, 308 (82.1%), 40 (10.6%), and 30 (7.4%), estimated samples were selected from each stratum Hawassa, Wolayta Soddo, and Dilla, respectively. All members of the selected bureau/office (clusters) were included in the survey until the required proportional number is reached.

### 2.5. Data Collection and Procedure

The tool used to collect data was LASA (Longitudinal Aging Study Amsterdam) SB Questionnaire. It was administered by five trained professionals. LASA SB Questionnaire is a self-administered questionnaire used to assess SB of older persons which contains 10 questions that require to respond the average time spent sitting per 24 hours on a weekday and weekend day. It requests respondents to report the duration of time spent in different described SBs, such as napping; reading; listen to music; watch television; watch video or DVD; perform a hobby; talk with friends, family, or acquaintances; sit at the computer for work or leisure; perform sitting activities such as administrative tasks; writing a letter or having a meeting; sit in car, bus or train, and on motorbike; visit church or (movie) theatre; sitting for meals per a day [31]. LASA comprises important behaviours like “visit church or (movie) theatre” which can be widely practiced by the population of the study, however, there are some important SB missing in LASA questionnaires such as “mailing hour,” “total sleeping hours per a day” (that can use to estimate correct waking time) but other sit questions consisted [13] were incorporated. Also, TV time is separated from video and DVD time and stands alone aimed to see particularly its prevalence comparing with existing evidence. Respondents were award ahead of the survey that the total sum of sitting hours in mentioned activities per day must not be greater than 24—sleeping hours + MVIPA time performed not less than 10 minutes [15, 32, 33].

Concerning reliability and validity, Visser & Koster reported that the mean total self-reported sedentary time was 10.4 (SD 3.5) hour/day and was not significantly different from the mean total objectively measured sedentary time (10.2 (1.2) hour/day, *p* ≤ 0.53). Total self-reported sedentary time on an average day (sum of twelve activities) correlated moderately (Spearman's *r* ≤ 0.35, *p* ≤ 0.01) with total objective sedentary time. The correlation improved when using the sum of six activities (*r* ≤ 0.46, *p* ≤ 0.01) and was much higher than when using TV watching only (*r* ≤ 0.22, *p* ≤ 0.05). The test-retest reliability of the sum of six sedentary activities was 0.71 (95% CI 0.57-0.81) [34]. Before delivery of survey, LASA questionnaire was translated from English to Amharic (the official language of the participant) and was done by existing language expertise in Dilla University.

The data collecting procedure was manual and direct contact with the participant. LASA Questionnaires' were distributed and collected contacting each sample bureau/office face to face in their office in the working days with the help of trained sport science professionals. Informed consent was obtained from each office/bureau head and the participant before conducting survey, and participation was voluntary and confidential. Also, ethical approval for the study was obtained from Dilla University Institutional Ethical Committee with Ref. No: DU/1-1/EM/-8/1513. The response and completion rates were 83% and 95%, respectively.

### 2.6. Assessment of Sedentary Time

The sedentary time of participants on weekdays and weekend days was assessed by using a self-reporting LASSA SB questionnaire consisting of 12 sedentary activities on weekdays and weekend days. Average sedentary hours across all days were calculated using a weighted average: (weekday hours × 5) + (week − end hours × 2)/7” [35]. The sum total of average sedentary hours spent in each (12) sedentary activities constitutes the total hours spent sedentary per day. The total sum of sitting hours in mentioned activities per day must not be greater than 24—sleeping hours + MVIPA time. IPAQ (International Physical Activity Questionnaire) data processing guidelines supporting only values of 10 or more minutes of activity will be included in the calculation of summary scores of PA or activities performed less than 10 minutes as of no use or considered sedentary [15, 32, 33]. According to Sloan et al., there are three levels, called “low,” “middle,” and “high” corresponding to (2.5 h/day), (5 h/day), and (10 h/day), respectively, determine sedentary level [16]. Even though there is limited and varied suggestion to determine a cut point hour for level of sedentary, in this study, we used cut-points for levels were less than 4 h/day, 4—less than 8 h/day, 8 to less than 11 h/day, and 11 or more h/day subsequently for “low,” “middle,” “high,” and “very high” were determined on the bases of Van der Ploeg et al. and Dunstan [36, 37]. Waking hours per day are determined by reducing sleeping time reported from 24 hours and the sum of computer time, TV time, and video/DVD/VCD time collectively constitute screen time.

### 2.7. Data Analysis

Statistical tests were performed using the program IBMSPSS Statistics version 20 (IBM Corporation, USA). Overall frequency distributions of demographic variables characteristics of the study subjects were examined to determine the estimated overall and sedentary time in each activity. Also, the time spent in each of 12 SA which were rated or presented in descending order from highest to lowest SB was identified. Waking time was computed by subtracting a sleeping hour from 24 and the percentage proportionality of time spent on a variety of sitting activity to describe the magnitude of activity within the list of activities. Independent *t*-test was performed to compare the mean of sitting time between males and females. Univariate ANOVA was conducted, and the effect of independent categorical variables (gender (G), education (E), marital status (MS), occupational responsibility (OR), and residing town (R)) on sitting time was examined. A directional relationship between demographic interval/ratio variables and multiple SB was run using a Pearson product-moment correlation. All reported *p* values were two-tailed, and statistical significance was set at 0.05 levels.

## 3. Results

### 3.1. Demographic Characteristic of the Respondents

In this study, a total of 375 urban civil servants working in various governmental offices in the eastern part of SNNPR, Ethiopia, were recruited as study participants. Sex, age, height, weight, education, income, marital status, responsibility, and residence were considered (IV). From the total number of respondents, 59.1% (222) and 40.9% (153) were men and women, respectively. Age category was 18–30, 31–40, 41–50, and 51–65 years old [12]. The highest proportions of individuals were in the age category of 31–40 years (36.1%), followed by those aged 18–30 years (26.7%). People in the age group 51–65 years made up 10.7% of the total sample. Education status was categorized into four groups (high school and below, college diploma, degree, masters, and Ph.D. and above). Majorities (63.4%) were degree/undergraduate; 12.6% had postgraduate education and office workers with a high school level education or below made up 6.7% of the total sample. On the basis of monthly earnings, nearly half (47.9%) earned a medium-income (between 3,000–4,999 birr/month). Those who earned a high (above 5,000 birrs/month) constituted 29.4% of the study population, while 22.7% were earning below 3,000 birr/month (low-income group). By marital status, 68.2%, 28.1%, and 1.6% of study participants were married, single, or divorced, respectively, while 2.1% fell outside of these three categories. Occupational responsibility was categorized under three headings: group/team leader, technical, professional, and nontechnical staff. Accordingly, the majority of respondents (82.6%) were professionals. The residence was categorized on the basis of the geographical location/towns. Hence, 82.4%, 10.4%, and 7.2% of respondents resided in Hawassa, Wolayta Soddo, and Dilla, respectively.

### 3.2. Prevalence of SB

Overall, descriptive statistics of SB were presented in Table 1. The majority of wake time (13.3869) h per day (about 80.1%) was spent sedentary. Waking time was found (16.7190) which is 24 h–7.2810 h (average sleeping h) + PA time. Among the 12 sedentary activities, the most prevailing was screen time (6.0781 h) which comprises computer time (3.1960 h), TV time (2.0781 h) and video, and DVD time (0.8039). Administrative task (1.4790 h), reading (1.3960 h), mealtime (1.0913 h), talking time (1.0473 h) per day revealed the second high prevalent SB. Worshipping hours, music time, transport time, napping hours, and hobby time accounted 0.8231, 0.6140, 0.6012, 0.1771, and 0.0799 h, respectively, were activities performed in a lower rate. Computer time and TV time are the most prevailing SB in which the majority of office workers were dependent on.

### 3.3. Gender Difference in Sitting Time

Activities performed while sitting/reclining by both sex was described in Table 1, and Independent *t*-test was computed to compare mean sitting time between male and female illustrated in Table 2. The result revealed that women spent much time than men in behaviours like screen time (6.4134–5.8470), meal time (1.183–1.0261), worshipping (0.8547–0.8013), and napping (0.0957–0.0690), respectively, where men were more involved in activities like administrative task (1.5554–1.3682), reading (1.512–1.1418), talk time (1.148), video/DVD (0.8270–0.7705), music (0.6465–0.5667), motor transport (0.7169–4332) than women, respectively, per a day. Statically computer time *t*(373) = −4.232, *p* < 0.00; reading time *t*(373) = 3.295, *p* < 0.001; meal time *t*(373) = −3.349, *p* < 0.001; transport time *t*(373) = 0.839, *p* < 0.000; and screen time *t*(373) = −2.333, *p* < 0.020 were significant.

### 3.4. Level of Sedentary Time

Sitting time was levelled in four categories described in Table 3, and found 70.7% of respondents were found to be very high sedentary, 23.7% were high sedentary, 4.8% were middle sedentary, and only 0.8% were low sedentary. Gender-wise, males accounted for the sum of 93.3% in a high and very high sedentary category within gender, whereas females were accounted for 96.1% within gender, which is higher than male. Generally, women reported higher sedentary time than men needs special concern. Nearly overall office working civil servants found to be very high sedentary is the alarming fact that needs due attention to carry out intervention.

### 3.5. The Relationship between Variables

Univariate ANOVA was conducted, and the effect of independent categorical variables (G, E, MS, OR, and R) on sitting time was examined. There was a statistically significant difference observed between *E* groups only *F* (3,371) = 7.649, *p* ≤ 0.000. A Tukey post hoc test revealed that time spent sitting by Masters was statically significantly higher than Degree holders (13.4398 ± 2.3 min, *p* ≤ 0.000) and (12.5618 ± 2.7 min, *p* ≤ 0.010), respectively. There was no statistically significant difference between high school and below and diploma holders (*p* ≤ 0.591) (see Table 4).

Directional relationship between demographic interval/ratio variables (age, income, and weight) and multiple SB was run using a Pearson product-moment correlation (Table 5). There was a weak positive correlation between age and time spent in administrative task, income, and meal time, which were statistically significant (*r* < 0.2, *n* = 375, *p* < 0.05). Meaning that, as age increases, administrative task also increases or with age decrease administrative task also decreases in the same direction. On the other hand, a weak negative relationship was observed between (age and talk time, screen time), (income and reading time), and (weight and mealtime), which were statistically significant (*r* < 0.2, *n* = 375, *p* < 0.05). This means that as one variable increases in value, the second variable decreases in value in the opposite direction.

## 4. Discussion

There are huge gaps in data or information in most African countries mainly in Ethiopia about the surveillance of PA and SB record trends [38]. The need for effective planning and policies addressing PA and SB [39, 40] should be based on scientific evidence, and it requires initiatives to deal with PA, SB. This study is the pioneer of its kind in the region or in the country that can provide comparative evidence on SB.

The findings of this study show that the majority of wake time per day was spent sedentary (16.7190 h). Sitting time was levelled in turtles and found 96.5%, 3.2%, and 0.3% are corresponding to high sedentary, middle, and low sedentary. Among the 12 sedentary activities, the majority of waking hours was spent collectively by screen time. Generally, women were reported to have a higher sedentary time than men, subsequently, 97.4% and 95.9%. Women spent much more time than men in SA like screen time, mealtime, worshipping, and napping, whereas men were more involved in activities like administrative tasks, reading, talk time, video/DVD, music, and motor transport than women per day.

Prevalence estimates or other necessary evidence on SB PA of adult's civil servant in the study region or in overall country is scarce to compare it with present finding, but contemporary researches reported prevalence estimates of SB PA among adults in country level were ample. The present prevalence estimates among office working civil servants are quite higher than the reports from different developed countries. A review of the adult's prevalence of sedentary among 5 Arabian Gulf region countries revealed that 61.0% of males and 73.7% of females were sedentary [11]. Spittaels et al. reported that 57% of US (7.89 h/a day), 55% of Sweden (7.7 h/a day), 57% of Australian (8.12 h/a day), 58% of European adults (8.12 h/a day), and 58% of western countries (7.89 h/a day) dedicated their waking time in sedentary pursuit. Men accumulate many steps per day than women [24]. The majority of Canadian adults waking hours 68% (9.6 h/day) for men and 69% (9.8 h/day) for women were sedentary [41]. As Dunstan et al. cited in [37], mix of working and nonworking Australian adults spent (60%) 9.3 h/day and accelerometer measured sedentary patterns of office workers work hours identified 75.8% of working h/day. Also, the recent report from Ergotron revealed Americans are sitting an average of 13 hours a day and sleeping an average of 8 hours resulting in a sedentary lifestyle of around 21 hours a day which is a similar trend with present finding [42]. As we can see from the previous literature, the prevalence estimate is on country or continental region level, which comprises a number of dissimilar groups that can include more sedentary or active diversified groups, which can moderate the result and time spent sedentary was lay in between 7 to 10 h per day. But the subjects of this study were a specific group office workers that were supposed to sit much time in the work office [43], expected relatively higher sitting time than other groups, and as a result, the estimate was found to be higher 80.1% of waking time (more than 13 h/day) compared with previous evidence. Because of population, groups that are most at risk of prolonged sitting include those working in offices, transportation, and highly mechanized trades [37]. Another important justification for elevated sitting time is, as it has been discovered by [44], those who sit for longer at work are more likely to sit outside of work or leisure time, so that office worker whose activity is more of computer use, writing, reading usually spent much time sedentary. Moreover, the most common and popular practice or culture in the study area and all over the country is office workers (civil servants) are expected to participate in different social sedentary activities, which are not incorporated in this study such as groaning, social congregations, and visiting bed waiters in their spare time that can add to their elevated daily sitting time. Even though 13 h/a day is a relatively higher level of sitting time, still shreds of evidence support the result of this study or even more than 13 h/a day reported in the present decade. For example, Ergotron witnessed global studies show people sit up to 15 hours a day, on average [12].

Female high level of sedentary time compared with men revealed in this study is a consistent trend with the previous estimate despite some figure variations [9, 10]. The overall sedentary time of women is higher than men in any country still goes the same, and no evidence appeared to excel opposing this trend so far.

Screen time (the time spent watching television and movies, playing video games, and using computers) is the leading SB among others. It accounts for the majority of share or almost about half of waking time (6.0781 h) spent sedentary indicated in the present findings is similar with the report of [9, 10]. BGR media report, daily distribution of screen minutes across 30 countries including some African countries such as South Africa (7.18 h/day), Kenya (6.73 h/day), Nigeria (7.38 h/day), and Saudi (7.38 h/day) spent time in front of screen [45]. Also eMarketers reported collectively screen time (excluding computer time) in the US on average 4 : 39 for watching live TV, 0 : 25 for DVR, and 0 : 11 for DVD that adds up to 5 : 15 minutes a day spent sedentary [27]. If computer time for work, internet time, and the likes, added time spent on the screen will be higher than the figure of the present finding. According to BGR media report, people of the United States is the sixth-worst nation who spend an average of 444 minutes (7.4 h) every day looking at the screens that breaks down to 147 minutes spent watching TV, 103 minutes in front of a computer, 151 minutes on smartphones, and 43 minutes with a tablet. At the top of the list is Indonesia, where people spend an average of 540 minutes or (9 h) each day looking at the screen [45]. As we can observe, computer use time become similar in all over the world, but comparatively, the present finding is a bit lower than the existing data. However, screen time is rising at in fast rate as can be speculated. This can be evidence that the influence of enhanced technology is not only affecting developed countries but also it is raising in a very fast rate in developing countries. The widespread availability of computers and labour-saving devices has risen the amount of sedentary time in recent decades [43] as speculated by researchers was quite right.

US adults spend online on desktop and laptop computers, in 2010, (2 : 22 h/day), in 2011, (2 : 33 h/day), in 2012, (2 : 27 h/day), and in 2013, (2 : 19) h/day on average [45]. Evidences are inconsistent; however, the present finding is a bit higher in comparison. Computer and computer use were not this much adequate or familiar before certain decades in the country level, but within a few years, no offices exist without a computer all over the country, and today, office work has become dependent of computer. In these transitional or transformational decades, computer use will increase dramatically in the country because manual systems are replacing, and interring data into a computer may take time, adapting computer use is a new custom, and communications are relay on computers. Due to low developed skill, operating computer may take much time for fewer tasks, poorer connections may cost much time. Moreover, due to the lack of smartphones and tablets, using a computer instead is an obligatory option and the likes can add to elevated time for computer use.

Regarding TV time, there are number of evidences available to compare TV time separate from screen time to compare with the present study. TV time is found to be the second-highest time next to computer use and it accounted for 12.4% of waking hours (2.0781 h/day) in this study. An article stated that US adults spent an average of 11 hours and 49 minutes with media each day in 2012 and forecasted 12 hours and 05 minutes with media in 2013 [45]. The US media “emarketer” reported the average time US adults spent watching video programming on TV totalled 4 hours, 35 minutes/day in 2011, 4 : 38 in 2012, 4 : 31 in 2013, 4 : 22 in 2014, and forecasted decline to 4 hours, 15 minutes in 2015 [46]. Another report by David Hinckley in New York daily news held on Wednesday, March 5, 2014, 5:27 PM revealed that the average American watches 5 hours of live TV per day and TV time increases steadily as they get older [47]. The Irish Times on its part reported “Irish adults aged 15 or older watched the small screen for an average of 3 hours and 28 minutes each day;” this figure does not include time spent on watching DVDs, online catch-up players, or so-called “over-the-top” services such as Netflix [48]. Hence, all the existing evidence about TV time from literature is greater than the present study finding of TV time. The reason behind this is unclear but reporting TV distinguished from time from DVD or VCD and the likes is not a familiar trend of the study population. This might be because of habit or practice in or there is no controlled recording diary or device just as developed countries. Moreover, except office work planning, time planning for such TV, Video, DVD, and the likes is not usual practice so that self-reporting time per each sitting activity may not be convenient to us to report actual time spent in a particular activity. In addition, most of Ethiopians have not developed the behaviour of recording daily diary for activities or regular practice trend for activities rather they perform activities instinctively as they got the opportunity to do. Despite all these limitations, an attempt to search and identify the sitting time of every daily activity is a must to help public society to be protected from the deleterious health effect of usually costumed trends like sitting too much. Researchers amplified TV time effect by forwarding warnings saying “Every hour spent watching television shortens the viewer's life by 22 minutes,” academics warn. “Anyone who spends six hours a day in front of the box is at risk of dying five years sooner than those who enjoy more active pastimes, it is claimed” [7]. This means that six hours daily sitting reduces our life span in five years, and as time increased sitting, life span decreases called negative relationships in between. Ergotron stated in this regard, the more you sit, “the poorer your health and the earlier you may die, no matter how fit you are” [12].

Also, time spent watching video on digital devices, PCs, mobile devices, and other connected devices including over-the-top and game consoles reported was totalled 21 minutes daily in 2011, 36 minutes in 2012, 50 minutes in 2013, 1 : 03 h in 2014, and in 2015. US adults spend an average of 1 hour, 16 minutes each day with video on digital devices [12]. The time estimated was in between 21 minutes and 1 : 16 minutes in which the present finding DVD Video time (0.8039 ≈ 0.48 minutes) lay in the range, meaning that the time is not much different from existing trend or consistent with contemporary study.

Concerning reading time, NOP World announced results of its Culture Score (TM) “Media Habits” Index offering a global perspective on the time consumers report watching television, listening to the radio, searching internet, and reading among 30 nations. Accordingly, hours spent on reading books around the world were estimated 8.9 h a week (1.27 h/day). US and UK are below the global average (0.81 and 0.76 h/day) but considerably above average in TV time (2.71 and 2.57 h/day), respectively. Of the 30 nations surveyed, India is the world number one most likely to spend time reading (1.53 h/day) and Koreans spent the least time reading: (0.45 h/day) [49]. The present finding (1.3960 h/day) can be levelled high time spent on reading in respect to global data, which needs farther investigation. However, offices work is characterized by rotten nonbook readings and workers are supposed to read plenty of letters, manuals, applications, and the likes on a daily basis may relatively put the subjects at higher reader level.

Transport time was found (0.839 h/day) dissimilar amount with previous studies, for example, Time Spent Travelling in Motor Vehicles (TSTMV) by Colombian adult were reported more than 120 minutes (2 h/day) [50], Americans the world highest owner of cars on average sit in their cars for 48 minutes each day, in Toronto, the average round trip commute time is 80 minutes [51].

Meal time is important time in sedentary study, and it was found to be 1.0913 h/day (6.5%) of waking time spent sitting for eating breakfast, lunch, and dinner. The average Britain's adult-only spends 23 minutes a day eating breakfast, lunch, and dinner. Britons are too busy to eat, even though they understood that they should spend at least 20 minutes eating each meal. Research indicates that they are in fact eating all three meals in a third of this time [52]. Survey responses of (2006-08), Americans age 15 or older spent 67 minutes in primary eating and drinking and additional 23.5 minutes were spent eating while doing something else totalled (1 : 35), 90 : 5 minutes/day [53].

Sedentary research in the country is scarce or nil, and this might be the pioneer research attempted to distinguish the distribution of sedentary pursuit among office-based workers. Comparative data within the country is deficient in which the strength and weak side of this research can be evaluated. Another important constraint to be mentioned is the subjective nature of self-report approach used to collect data may be associated with some of overreporting high time or under reporting low time bias due to lack of recorded pattern of diary, or recall bias and its inherited likelihoods of errors. However, cross-sectional study is still having considerable universal acceptance for such study [54]. Indeed, it is impossible to generalize the result of this finding to other urban dwellers except office workers in the country. On other hands, it paves new ground that can trigger farther question or research. It also provides comparative data in sedentary research in the country. The use of appropriate tools and representative samples, prioritizing the major task forces, identifying the most victimized group of the society and the likes, can be mentioned as strength of this research.

## 5. Conclusion

This study provides overall time spent on SA in office and out of office in waking hours and accordingly, over ¾ of waking time is spent on sitting. Screen time shared about ½ of total sitting time, and women are found to be highly sedentary than men. Sedentary time and its associated effect have been increasingly acknowledged in office-based work, and the high level of self-reported sedentary time record suggests the need for public health policies targeted at increasing physical activity and decreasing sitting time through systemic intervention in and out of work. Responsible bodies should support and facilitate the reduction of sitting too much time in the workplace. A higher level of sitting time was seen in women than men is a considerable homework for public health policy because increasing PA is a societal, not just an individual problem. Therefore, according to WHO suggestion, intervention demands a population-based, multispectral, multidisciplinary, and culturally relevant approach. There is not much evidence or data are limited in Africa about SB particularly in Ethiopia. Research's addressing SB in old age, children, and rural dwellers is highly essential. Finally, it will be important to measure the magnitude of the practice of SB periodically to see if it changes over time.

## Figures and Tables

**Table 1:** Descriptive statistics of average SB per day.

Rank	Sedentary activities performed per a day	Group statistics					% of wake hour
		Gender	*N*	Mean hours	Std. deviation	Std. error mean	
1	Computer time	Male	222	2.8979	1.66326	0.11163	19.1%
		Female	153	3.6286	1.61062	0.13021	12.4%
	Average computer time		375	3.1960	1.67878		19.1%
2	TV time	Male	222	2.1221	1.21758	0.08172	8.8%
		Female	153	2.0143	1.25226	0.10124	8.3%
	Average TV time		375	2.0781	1.23133		12.4%
3	Administrative tasks	Male	222	1.5554	1.34813	0.09048	6.5%
		Female	153	1.3682	1.32974	0.10750	6.3%
	Average admin time		375	1.4790	1.34204		8.8%
4	Reading hours	Male	222	1.5712	1.30258	0.08742	4.9%
		Female	153	1.1418	1.14408	0.09249	4.8%
	Average reading hours per day		375	1.3960	1.25667		8.3%
5	Meal hours	Male	222	1.0281	0.41842	0.02808	3.7%
		Female	153	1.1831	0.47062	0.03805	3.6%
	Average meal hours per a day		375	1.0913	0.44641		6.5%
6	Talking hours	Male	222	1.1486	0.87013	0.05840	1.1%
		Female	153	0.9004	0.82052	0.06633	0.48%
	Average talking hours per day		375	1.0473	0.85786		6.3%
7	Worshipping, theatre, cinema hours	Male	222	0.8013	0.62523	0.04196	19.1%
		Female	153	0.8547	0.56234	0.04546	12.4%
	Average worshipping hours		375	0.8231	0.60017		4.9%
8	Average time spent watching video, DVD	Male	222	0.8270	0.94715	0.06357	8.8%
		Female	153	0.7705	0.93508	0.07560	8.3%
	Average video, DVD time		375	0.8039	0.94140		4.8%
9	Average time spent listening music	Male	222	0.6465	0.66373	0.04455	6.5%
		Female	153	0.5667	0.74311	0.06008	6.3%
	Average music time		375	0.6140	0.69734		3.7%
10	Average time spent using motor transport	Male	222	0.7169	0.75293	0.05053	4.9%
		Female	153	0.4332	0.53255	0.04305	4.8%
	Average motor transport		375	0.6012	0.68538		3.6%
11	Average time spent performing hobbies	Male	222	0.1953	0.53270	0.03575	3.7%
		Female	153	0.1507	0.46242	0.03738	3.6%
	Average hobby time		375	0.1771	0.50504		1.1%
12	Average napping hours	Male	222	0.0690	0.14509	0.00974	1.1%
		Female	153	0.0957	0.23200	0.01876	0.48%
	Average napping hours per day		375	0.0799	0.18571		0.48%
Total average screen time (computer, TV^,^ video/DVD time) per day			375	6.0781	2.32464		36.4%
Average waking hours per day			375	16.7190	1.14994		100%
Total sitting hours per day			375	13.3869	2.73668		81.1%
Average sleeping hours per day			375	7.2810	1.14994		43.5%

**Table 2:** Gender difference in sitting time.

		Levene's test for equality of variances		*t*-test for equality of means						
		*F*	Sig.	*t*	df	Sig. 2 tailed	Mean difference	Std. error difference	95% CI of the difference	
									Lower	Upper
Computer time	Equal variances assumed	0.827	0.364	-4.235	373	0.000	-0.73066	0.17253	-1.06992	-0.3914
	Equal variances not assumed			-4.260	333.598	0.000	-0.73066	0.17151	1.06804	-0.39328
Reading hours	Equal variances assumed	1.355	0.245	3.295	373	0.001	0.42940	0.13034	0.17312	0.68569
	Equal variances not assumed			3.374	351.791	0.001	0.42940	0.12727	0.17910	0.67971
Meal hours	Equal variances assumed	0.000	0.990	-3.349	373	0.001	-0.15496	0.04628	-0.24596	-0.06396
	Equal variances not assumed			-3.277	301.238	0.001	-0.15496	0.04729	-0.24802	-0.06191
Screen time	Equal variances assumed	3.081	080	-2.333	373	0.020	-0.56641	0.24282	-1.04388	-0.08894
	Equal variances not assumed			-2.373	345.265	0.018	-0.56641	0.23873	-1.03596	-0.09686
Transport time	Equal variances assumed	9.923	0.002	4.019	373	0.000	0.28371	0.07060	0.14489	0.42253
	Equal variances not assumed			4.274	372.736	0.000	0.28371	0.06639	0.15317	0.41425

**Table 3:** Sedentary category/level.

Gender of respondents ∗ sedentary category cross-tabulation							
			Sedentary category				Total
			Low sedentary	Middle sedentary	High sedentary	Very high sedentary	
Gender	Male	Count	2_a, b_	13_a, b_	42_b_	165_a_	222
		% within gender	0.9%	5.9%	18.9%	74.3%	100.0%
		% within sedentary category	66.7%	72.2%	47.2%	62.3%	59.2%
		% of Total	0.5%	3.5%	11.2%	44.0%	59.2%
	Female	Count	1_a, b_	5_a, b_	47_b_	100_a_	153
		% within gender	0.7%	3.3%	30.7%	65.4%	100.0%
		% within sedentary category	33.3%	27.8%	52.8%	37.7%	40.8%
		% of total	0.3%	1.3%	12.5%	26.7%	40.8%
Total		Count	3	18	89	265	375
		% within gender	0.8%	4.8%	23.7%	70.7%	100.0%
		% within sedentary category	100.0%	100.0%	100.0%	100.0%	100.0%
		% of total	0.8%	4.8%	23.7%	70.7%	100.0%

Each subscript letter denotes a subset of sedentary category categories whose column proportions do not differ significantly from each other at the.05 level.

**(a):** 

ANOVA					
Average total sitting time					
	Sum of squares	df	Mean square	F	Sig.
Between groups	163.158	3	54.386	7.649	0.000
Within groups	2637.878	371	7.110		
Total	2801.036	374			

**(b):** 

Multiple comparisons						
Dependent variable: average total sitting time Tukey HSD						
(I) Educational status	(J) Educational status	Mean difference (I-J)	Std. error	Sig.	95% confidence interval	
					Lower bound	Upper bound
High school and below	Diploma	-0.78888	0.62753	0.591	-2.4084	0.8306
	Degree	-1.75869^∗^	0.56061	0.010	-3.2055	-0.3119
	Masters	-2.63661^∗^	0.66007	0.000	-4.3400	-0.9332
Diploma	High school and below	0.78888	0.62753	0.591	-0.8306	2.4084
	Degree	-0.96980^∗^	0.37318	0.048	-1.9329	-0.0067
	Masters	-1.84772^∗^	0.51056	0.002	-3.1653	-0.5301
Degree	High school and below	1.75869^∗^	0.56061	0.010	0.3119	3.2055
	Diploma	0.96980^∗^	0.37318	0.048	0.0067	1.9329
	Masters	-0.87792	0.42562	0.167	-1.9763	0.2205
Masters	High school and below	2.63661^∗^	0.66007	0.000	0.9332	4.3400
	Diploma	1.84772^∗^	0.51056	0.002	0.5301	3.1653
	Degree	0.87792	0.42562	0.167	-0.2205	1.9763

^∗^The mean difference is significant at the 0.05 level.

**Table 5:** Pearson correlation for age, income, weight vs. SB.

Correlations			
		Age of respondents	Average time spent performing administrative tasks per a day
Age of respondents	Pearson correlation	1	0.132^∗^
	Sig. (2-tailed)		0.011
	*N*	375	375
Average time spent performing administrative tasks per a day	Pearson correlation	0.132^∗^	1
	Sig. (2-tailed)	0.011	
	*N*	375	375
Income of respondent	Pearson correlation	1	0.141^∗∗^
	Sig. (2-tailed)		0.006
	*N*	375	375
Average meal hours per a day	Pearson correlation	0.141^∗∗^	1
	Sig. (2-tailed)	0.006	
	*N*	375	375
Age of respondents	Pearson correlation	1	-0.149^∗∗^
	Sig. (2-tailed)		0.004
	*N*	375	375
Average talking hours per a day	Pearson correlation	-0.149^∗∗^	1
	Sig. (2-tailed)	0.004	
	*N*	375	375
Income of respondent	Pearson correlation	1	-0.125^∗^
	Sig. (2-tailed)		0.015
	N	375	375
Average reading hours per a day	Pearson correlation	-.125^∗^	1
	Sig. (2-tailed)	.015	
	*N*	375	375
Weight of respondents	Pearson correlation	1	-0.129^∗^
	Sig. (2-tailed)		0.012
	*N*	374	374
Average meal hours per a day	Pearson correlation	-0.129^∗^	1
	Sig. (2-tailed)	.012	
	*N*	374	375
Age of respondents	Pearson correlation	1	-0.117^∗^
	Sig. (2-tailed)		0.024
	*N*	375	375
Total average screen time	Pearson correlation	-0.117^∗^	1
	Sig. (2-tailed)	.024	
	*N*	375	375
Weight of respondents	Pearson correlation	1	-0.129^∗^
	Sig. (2-tailed)		0.012
	*N*	374	374
Average meal hours per a day	Pearson correlation	-0.129^∗^	1
	Sig. (2-tailed)	0.012	
	*N*	374	375

^∗^Correlation is significant at the 0.05 level (2-tailed).

## Data Availability

The data used to support the findings of this study are available from the corresponding author upon reasonable request.

## Abbreviations

ACSS: American Cancer Society Study
BPS: British psychological society
DV: Dependent variable
DVD: Digital versatile disc
HDL: High-density lipoprotein
IV: Independent variable
IPAQ: (International Physical Activity Questionnaire)
LASA: Longitudinal Aging Study Amsterdam
LPL: Lipoprotein lipase
METs: Metabolic equivalent times
MIPA: Moderate-intensity physical activity
MOSHE: Ministry of Science and Higher Education
MVIPA: Moderate vigorous-intensity physical activity
NCDs: Noncommunicable diseases
OR: Organizational responsibility
PA: Physical activity
PhD: Doctor of philosophy
SB: Sedentary behaviour
SD: Standard deviation
TSTMV: Time spent travelling in motor vehicles
SNNPR: South nation nationality people region
TV: Television
UK: United Kingdom
US: United States
USA: United States of America
VCD: Video compact disc
VIPA: Vigorous-intensity physical activity.
